# Diagnostic Potential of Circulating miRNAs in Glioma: A Systematic Review and Meta-Analysis

**DOI:** 10.3390/ijms27041680

**Published:** 2026-02-09

**Authors:** Aizere Khassenova, Zhamilya Seitkanova, Alissa Loskutova, Rostislav Bukasov, Olena Filchakova

**Affiliations:** 1 Biology Department, School of Sciences and Humanities, Nazarbayev University, Kabanbay Batyr Ave., 53, Astana 010000, Kazakhstan; aizere.khassenova@nu.edu.kz (A.K.); zhamilya.seitkanova@nu.edu.kz (Z.S.); 2 Chemistry Department, School of Sciences and Humanities, Nazarbayev University, Kabanbay Batyr Ave., 53, Astana 010000, Kazakhstan; alissa.loskutova@nu.edu.kz (A.L.); rostislav.bukasov@nu.edu.kz (R.B.)

**Keywords:** glioma, glioblastoma, miRNA, biomarkers, diagnostics

## Abstract

Gliomas are intracranial tumors characterized by limited diagnostics and treatment approaches. Blood-circulating miRNAs represent a regulatory class of molecules that change their expression under pathological conditions and can relatively easily be detected. The present study evaluates the diagnostic potential of blood-circulating miRNAs in glioma. All grades of gliomas are included in the analysis. The articles were retrieved from the PubMed, Web of Science and Scopus databases up to October 2025. The studies were considered to be eligible if they used glioma patients and healthy controls and compared their miRNA levels, indicating sensitivity and specificity values. Risk of bias was assessed using the QUADAS-2 tool. The collected data was pooled by the STATA 19.0 MP bivariate random effects model and indicated heterogeneity using the I^2^ statistic value. To identify possible reasons for heterogeneity, we utilized subgroup analysis and meta-regression. Publication bias was assessed with Deeks’ funnel plot, and the test diagnostic potential was evaluated with Fagan’s nomogram. We analyzed 31 original reports covering 2299 glioma patients and 1719 healthy controls. A meta-analysis on 59 data points extracted from the analyzed papers was conducted. The combined pooled sensitivity was found to be equal to 0.83 (95%CI: 0.80–0.86), the specificity 0.88 (95%CI: 0.85–0.90), the positive likelihood ratio 6.7 (95%CI: 5.4–8.5), the negative likelihood ratio 0.19 (95%CI: 0.16–0.23), and the diagnostic odds ratio 35 (95%CI: 25–50). An SROC analysis revealed an AUC equal to 0.92 (95%CI: 0.90–0.94). The reported diagnostic parameters imply that blood-circulating miRNAs hold the potential to be developed into diagnostic biomarkers for glioma identification. However, the high heterogeneity in the analyzed studies suggests that the results should be considered as exploratory only.

## 1. Introduction

Gliomas are brain tumors that originate from neural stem cells that have acquired tumorigenic mutations [1]. Gliomas are classified according to the criteria outlined in the fifth edition of the WHO Classification of Tumors of the Central Nervous System (CNS) published in 2021 [2]. Gliomas can be of either adult type or pediatric type depending on the age of an affected person. Adult-type diffuse glioma is by far the most common type, which accounts for more than 90% of cases. It includes three types of gliomas: astrocytoma (IDH-mutant), oligodendroglioma (IDH-mutant and 1p/19q-codeleted), and glioblastoma (IDH-wildtype).

The current diagnosis of glioma is based on brain imaging, followed by histological and molecular biomarker characterization of a tumor sample [2]. Histological features based on cellular differentiation enable determining the type of glioma (e.g., astrocytoma or oligodendroglioma). Histological anaplastic features that include necrosis, proliferation of microvasculature, and mitotic activity enable determining the tumor grade.

Gliomas are classified into four grades (I–IV). Grade I gliomas usually have a good prognosis and are characterized by well-demarcated tumors. The most frequent example of Grade I glioma is pilocytic astrocytoma, usually diagnosed in children or adolescents. Grade II gliomas can demonstrate brain invasion, and thus their complete resection might be difficult, which contributes to a poorer prognosis. Both Grade I and II gliomas are characterized by slow growth and are considered to be low-grade gliomas (LGG). Grades III and IV gliomas are characterized by rapid aggressive growth and poor patient outcomes, with Grade IV having the worst prognosis. Both Grade III and IV demonstrate anaplastic histological features and are considered to be high-grade gliomas (HGGs). The most common gliomas in adults, adult diffuse gliomas, belong to Grades II to IV, with oligodendroglioma (IDH-mutant and 1p/19q-codeleted) representing Grades II–III, astrocytoma (IDH-mutant)—Grades II–IV, and glioblastoma (IDH-wildtype), the most aggressive and common primary malignant brain tumor in adults, belonging to Grade IV glioma. According to the gene expression profile, GBM is classified into proneural, neural, classical, and mesenchymal subtypes [3]. The classical subtype is defined by chromosome 7 amplification and chromosome 10 loss, together with aberrations in the gene expression of EGFR (Epidermal Growth Factor Receptor). Mesenchymal GBM is characterized by low expression of NF1 (Neurofibromin 1) due to the hemizygous deletion of the 17q11.2 region that contains the *NF1* gene. The proneural type is characterized by alteration in PDGFRA (Platelet-Derived Growth Factor Receptor Alpha) together with mutations in *IDH1* and *TP53* genes. The neuronal subtype demonstrates expression of neuronal markers, such as NEFL (Neurofilament Light Chain), GABRA1 (gamma-aminobutyric acid receptor α1), SYT1 (Synaptotagmin-1), and SLC12A5 (Solute Carrier Family 12 Member 5). Glioblastoma could be either primary, originating spontaneously without prior history of low-grade glioma, or secondary, arising from previous low-grade glioma [4].

The molecular biomarkers that are currently incorporated into glioma diagnostics include mutation within the isocitrate dehydrogenase (*IDH*) gene [5]. The mutated *IDH* gene is correlated with a better prognosis in glioblastoma [6]. Thus, it could be considered as a prognostic biomarker for glioblastoma. In addition to *IDH* mutations, other genes can be altered in gliomas. They include *ATRX*, *TP53*, *CDKN2A/B*, *CIC*, *FUBP1*, *NOTCH1*, and *EGFR* [2]. Alongside the mutations within genes, changes at the chromosome level, such as 1p/19q codeletion [7], and mutations within the *TERT* promoter [8] are considered as molecular markers for the diagnostics of adult diffuse gliomas. It is worth noting that all the listed molecular markers currently used for the diagnostics of adult diffuse gliomas are invasive, requiring tissue biopsy. This limits wide application of the above biomarkers, especially for early cancer detection.

With the currently available diagnostic tools that rely on histological and molecular characterization of tissue, gliomas are often detected at a late stage when the treatment options are limited and are not efficient. This highlights the need for the development of a robust low-invasive diagnostic approach that would rely on glioma-specific biomarkers during its earlier stages.

Liquid biopsy has emerged as a promising non-invasive approach for biomarker detection in bodily fluids, such as blood, cerebrospinal fluid, and urine [9]. Among the different classes of circulating biomarkers—such as circulating tumor cells [10], circulating free DNA [11], and extracellular vesicles [12]—microRNAs have gained increasing attention due to their stability, specificity, and functional relevance in cancer biology [13]. Because miRNAs are secreted into circulation directly or via exosomes [14], they have been widely investigated as potential blood-based biomarkers for glioma and glioblastoma diagnosis [15]. Compared to traditional protein-based biomarkers, circulating miRNAs offer several advantages: high stability: miRNAs are protected from RNase degradation by encapsulation in exosomes or association with proteins; and non-invasive detection: they can be detected in blood, CSF, and urine, making them accessible for routine liquid biopsy testing and early disease detection [15]. These properties make miRNAs ideal candidates for developing into a minimally invasive diagnostic tool for gliomas.

In the current review, we focus on the evaluation of blood-circulating microRNAs as potential biomarkers for glioma diagnostics. We collected and analyzed original publications that evaluated the expression levels of circulating miRNAs in glioma patients compared to healthy controls. Our goal was to investigate the potential of circulating miRNAs as non-invasive biomarkers for the diagnosis of gliomas. Low-grade gliomas as well as high-grade gliomas were considered. Our meta-analysis evaluates the diagnostic accuracy of biomarkers across the full glioma spectrum (all grades included) to identify candidates for early detection, thereby preventing progression to high-grade malignancy, such as glioblastoma. We collected original papers on adult gliomas only because adults have higher incidence of gliomas compared to children. The incidence for adult gliomas was reported at around 4.67–5.73 per 100,000 annually [16], while the incidence for pediatric gliomas was reported at 2.37 per 100,000 [17]. Moreover, the character of the gliomas differs: two thirds of pediatric gliomas are low-grade, while 80% of adults’ gliomas are high-grade gliomas [18].

## 2. Methods

### 2.1. Study Selection

A comprehensive literature search was conducted across multiple databases, including PubMed, Web of Science and Scopus, to identify relevant studies evaluating the diagnostic potential of miRNAs in glioma. The search strategy included combinations of the following key words: “miRNA”, “microRNA”, “glioma”, “glioblastoma”, “glioblastoma multiforme”, “GBM”, and exclusion of “exosomal miRNA”, “extracellular vesicles”, “long non-coding RNA”, “non-human studies” and “cell lines”. The meta-analysis was written according to the PRISMA guidelines. The study was registered on PROSPERO with reference number CRD420251053652.

### 2.2. Inclusion and Exclusion Criteria

**Figure 1:**
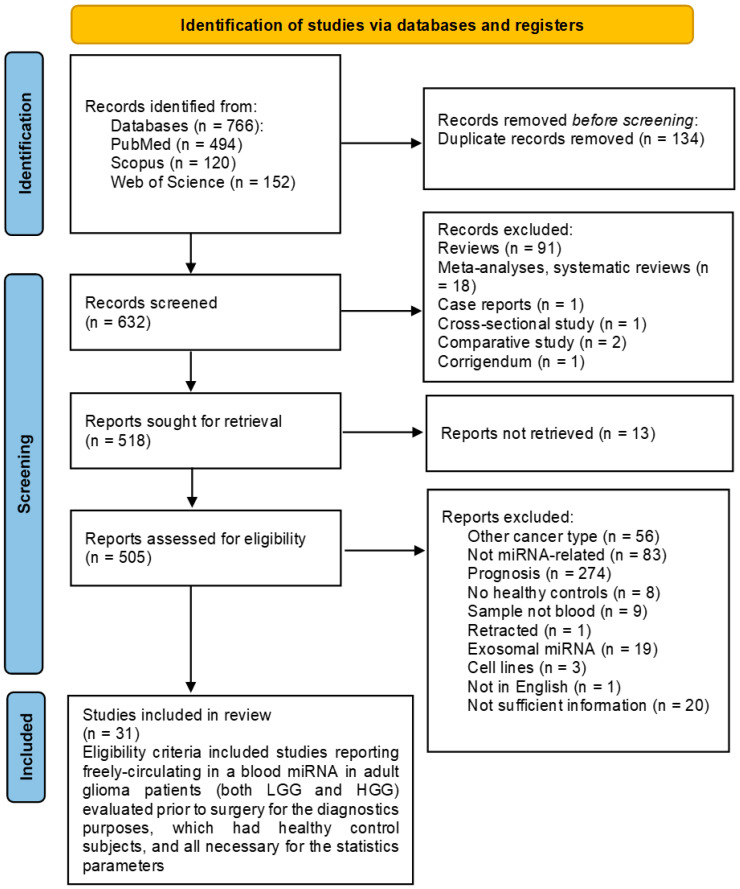
PRISMA 2020 flow diagram of the study selection process.

Studies were included if they met the following criteria: original research evaluating the expression of specific miRNAs in blood of glioma patients compared to healthy controls containing the sample size, number of glioma and healthy control patients, and sensitivity and specificity values. Exclusion criteria included non-miRNA-related studies, reviews, case reports, conference abstracts, duplicate studies, and non-English articles (Figure 1).

### 2.3. Study Sections and Data Extraction

The extracted data included author names, publication year, country of origin, journal where the articles was published, sample size, sample type, glioma grade, investigated miRNA, sensitivity, specificity, area under the curve, mode (upregulated or downregulated), and used method.

### 2.4. Quality Assessment

The Quality Assessment of Diagnostic Accuracy Studies-2 (QUADAS-2) tool was used to assess the quality of diagnostic studies, evaluating risk of bias in patient selection, index test, reference standard, and flow and timing. Heterogeneity among studies was assessed using Cochrane’s Q and the I^2^ statistic. An I^2^ value greater than 50% indicated significant heterogeneity, and potential sources of heterogeneity were explored using subgroup analysis and meta-regression based on study characteristics such as sample size (≥100; <100), detection method (RT-qPCR or microarray), mode (up/downregulation), sample type (plasma or serum), miRNA profile (single or cluster), glioma grade and study region (Asia and others).

### 2.5. Data Analysis

We used STATA MP 19.0 to analyze the data collected for meta-analysis. As the values of true positives, true negatives, false positives and false negatives were not reported in the articles, the sensitivity, specificity and sample size were used to calculate them manually in the following way: TP = (sensitivity) × (patient cohort); TN = (specificity) × (healthy cohort); FP = (1-specificity) × (healthy cohort); FN = (1-sensitivity) × (patient cohort). The resultant values are shown in Supplementary Table S1. Then, values such as pooled sensitivity, specificity, area under the curve (AUC), positive likelihood ratio (PLR) and negative likelihood ratio (NLR), and diagnostic odds ratio (DOR), along with their 95% confidence intervals, were calculated. The bivariate random effects model was used to build the summary receiver operator characteristic curve. Heterogeneity was evaluated using I^2;^ in case the value was >50%, it was considered significant. Fagan’s nomogram was built using PLR and NLR and pre-test probability to evaluate how the diagnostic test affects the diagnosis of the patient in case the test is positive or negative.

### 2.6. Publication Bias

Publication bias was evaluated using Deeks’ funnel plot asymmetry test for diagnostic studies by building a regression line across study observations and checking the slope and distribution of studies. A *p*-value of less than 0.05 was considered statistically significant.

## 3. Results and Discussion

### 3.1. Study Selection, Characteristics, and Quality Assessment

A literature search was conducted by two authors according to the keywords on Web of Science, PubMed and Scopus, and 766 articles were extracted. Indeed, 134 were found to be duplicates and removed before screening, and 632 reports were screened (Figure 1). Articles of no relevance, reviews, meta-analyses, and case studies were excluded from the study (n = 110). Ultimately, 518 reports were sought for retrieval, and 13 were not retrieved. Further, 505 reports were assessed for eligibility. The eligibility criteria included studies reporting freely circulating miRNAs in the blood of adult glioma patients (both LGG and HGG) evaluated prior to surgery for diagnostic purposes, which had healthy control subjects and all necessary parameters for statistical analysis. The reports were excluded if they dealt with other types of cancer or cell lines, did not include miRNA detection, dealt with prognostic potential of miRNAs rather than diagnostic, did not include healthy controls, detected miRNAs in exosomes or other non-blood bodily fluids, did not contain sufficient information for the meta-analysis, were retracted, or were written in non-English. Finally, 31 articles were included in the meta-analysis.

The quality assessment was done in accordance with QUADAS-2 criteria (Supplementary Figure S1) across four major domains: patient selection, index test, reference standard, and flow and timing. All included studies had a case-control setup; thus, the evaluation of the bias for patient selection was based on two remaining signaling questions. The bias for patient selection and index test was unclear for most of the studies. Most studies did not state explicitly whether a consecutive or random sample of patients was enrolled, leading to “unclear” risk of bias for the patient selection criterion. The major reason for the unclear risk of bias in the index test and reference standard domain was the absence of information about whether investigators were blinded to the results of the index test while performing the reference standard and vice versa. Regarding applicability concerns, most of the studies had low applicability concerns in patient selection, index test, and reference standard. In each study, the reference standard was chosen so that it correctly identifies glioma. 

The articles selected for the meta-analysis were published between 2012 and 2025 (Table 1).

The most prevalent country among the evaluated studies was China (22 papers). The other countries include Turkey (two papers), Egypt (two papers), and Italy, Hungary, USA, Japan, and Russia each contribute a single publication. Most of the articles (64.5%) used serum, while 11 articles (35.5%) had plasma as the sample source of microRNAs. The difference between serum and plasma samples includes clot formation that occurs in serum but is blocked by anticoagulants in plasma samples. It is possible that plasma and serum samples differ in terms of assessed microRNAs. At this point, it is difficult to decide which blood sample is preferable—serum or plasma. A contradiction exists in the literature where studies suggesting plasma as a better source of microRNAs [50] are opposed by studies showing more stable results over time for serum samples [51]. For example, Dufourd et al. [50] showed that rat plasma contains more miRNAs compared to serum, suggesting that plasma samples outperform serum ones. However, Wakabayashi et al. [51], while looking at the samples over time, found that 173 miRNAs showed a decline after collection from plasma, whilst no serum-derived miRNAs demonstrated time-course changes. Thus, more work is needed to compare glioma-related microRNA in serum vs. plasma samples. The inclusion of both plasma and serum samples in our study introduces a potential source of heterogeneity; however, at the same time, the inclusion of all the studies gives us a reliable sample size. In the future, with the accumulation of more data, it might be possible to evaluate serum-derived microRNAs and plasma-derived microRNAs separately.

The total number of patients in the analyzed papers was equal to 2299; the number of healthy controls was 1719.

Table 2 outlines the quantitative characteristics, such as sensitivity, specificity and area under the ROC curve (AUC), of the different miRNAs in the included studies. Overall, 59 data points were extracted from 31 publications for the analysis. Notably, the most promising microRNAs that show significant diagnostic potential are miR-106a and miR-200a, reported in Ali [19]; miR-21, miR-128, and miR-342-3p; reported in the Wang Q. paper [36]; and miR-221 [33]. The specificities for the above-mentioned miRNAs were reported to be equal to 1.0, while the sensitivities were ≥0.9. The lowest sensitivity was reported for miR-22-3p at 0.417 [20]; the lowest specificity was reported by Xu W. for miR-17 at 0.553 [41].

In the current work, we combined studies that evaluated the miRNA expression in patients with different types of gliomas with studies that looked specifically at glioblastoma only (Grade IV glioma). This approach aligns with previously published work [52,53,54,55]. Also, studies 10, 15–18, 20–22, 25, 45–52, 54, 55, 58, and 59 report on miRNA expression in patients with glioma of different grades, including Grade IV glioblastoma. Thus, it is practically impossible to stratify glioma Grades I–IV from Grade IV glioblastoma in such studies.

### 3.2. Meta-Analysis

#### 3.2.1. Diagnostic Accuracy of Circulating miRNAs in Glioma

**Figure 2:**
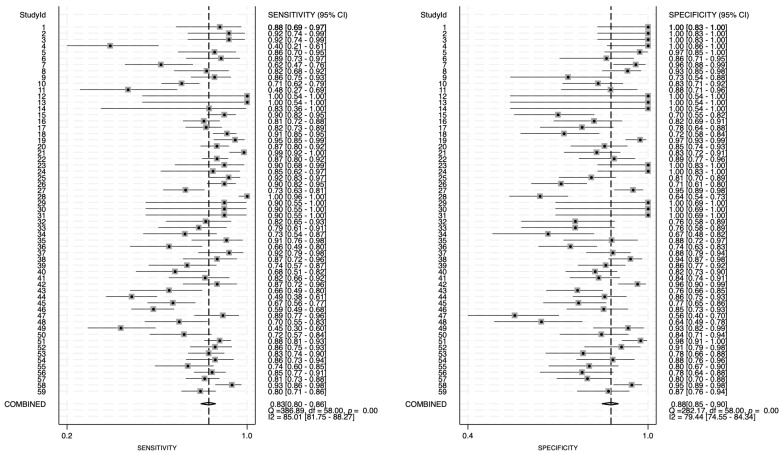
Forest plots for sensitivity and specificity values of analyzed papers. Thirty-one analyzed papers with 59 entries for miRNAs were analyzed. Pooled sensitivity and specificity and their heterogeneity were calculated using STATA MP 19.0 software.

The forest plot of the pooled sensitivity and specificity of circulating miRNAs in the diagnosis of glioma is shown in Figure 2. By pooling the forest plots, the heterogeneity is found to be 85.01% (95%CI: 81.75–88.27) and 79.44% (95%CI: 74.55–84.34) for sensitivity and specificity, respectively, which is an indicator of high heterogeneity. The combined estimation of the diagnostic accuracy of circulating miRNAs in glioma is as follows: sensitivity 0.83 (95%CI: 0.80–0.86); specificity 0.88 (95%CI: 0.85–0.90). The calculated accuracy is 0.828.

**Figure 3:**
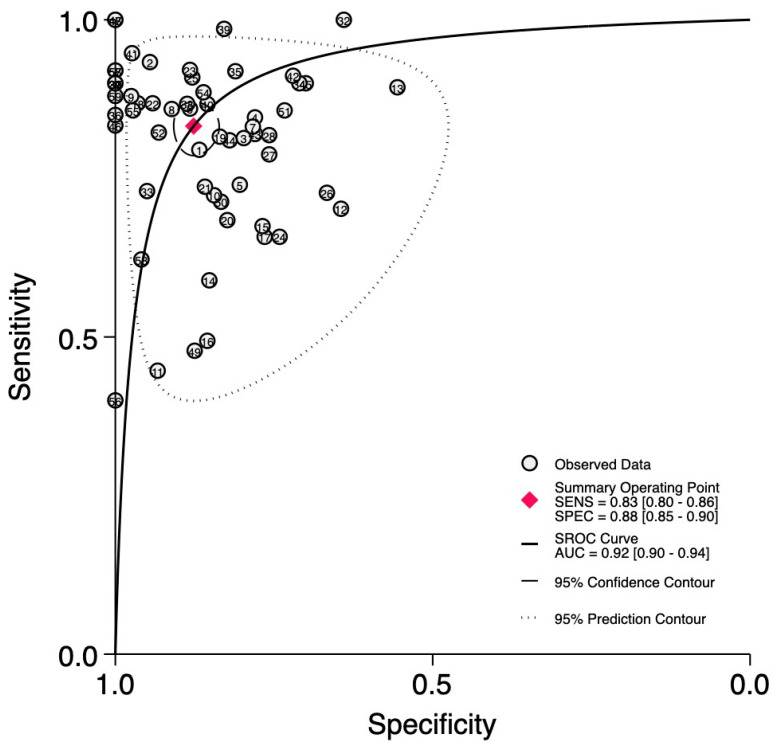
SROC curve of the diagnostic power of circulating miRNAs in glioma. Fifty-nine data points reported in 31 analyzed papers were used to build the graph using STATA software.

The SROC curve in Figure 3 did not exhibit a shoulder–arm-like distribution, suggesting that there is no threshold effect. The AUC was determined to be 0.92 (95%CI: 0.90–0.94). Such a value for AUC serves as an indicator of miRNA’s high diagnostic accuracy in glioma diagnosis.

Among all the included studies, the study by Géczi D. et al. [26] had the smallest sample size, with six patients and six control subjects. This led to the difference in derived values for sensitivity and specificity compared to the reported ones in entry #12–14 (Supplementary Table S1). Thus, this small-number study can potentially skew the results of pooled values. When this study was excluded from the analysis, the pooled sensitivity value became equal to 0.83 (95%CI: 0.79–0.86), pooled specificity value to 0.87 (95%CI: 0.84–0.90), and AUC remained at 0.92 (95%CI: 0.89–0.94) (Supplementary Figure S2).

**Figure 4:**
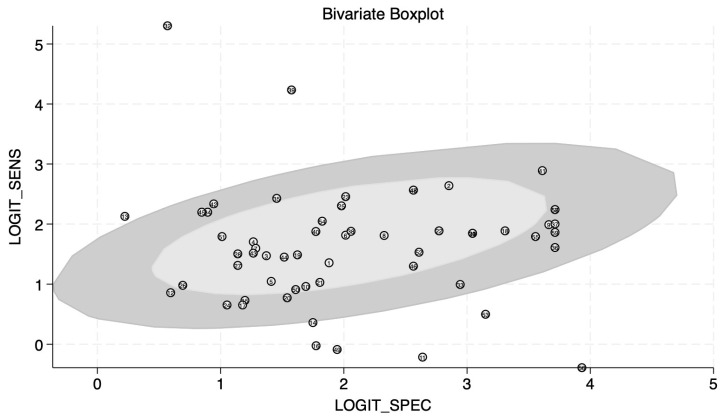
Bivariate boxplot of the selected studies. Out of 59 entries considered in the analysis, eight observations fall into “outlier” category.

The bivariate boxplot (Figure 4) represents the correlation and possible outliers among the studies used for the meta-analysis. The central ellipse shows studies whose values lie within the median and interquartile ranges. The outside ellipse shows data that lie within 95%CI; the studies outside of the outer ellipse are potential outliers. There are eight outlier observations noted in this review (entries #11, 13, 16, 32, 39, 49, 53, and 56). After excluding the outliers detected by the boxplot, the statistics become the following: sensitivity 0.84 (95%CI: 0.81–0.86), specificity 0.88 (95%CI: 0.85–0.91), PLR 7.0 (95%CI: 5.4–9.0), NLR 0.19 (95%CI: 0.16–0.22), DOR 38 (95%CI: 26–56), and AUC remained the same at 0.92 (95%CI: 0.89–0.94).

**Figure 5:**
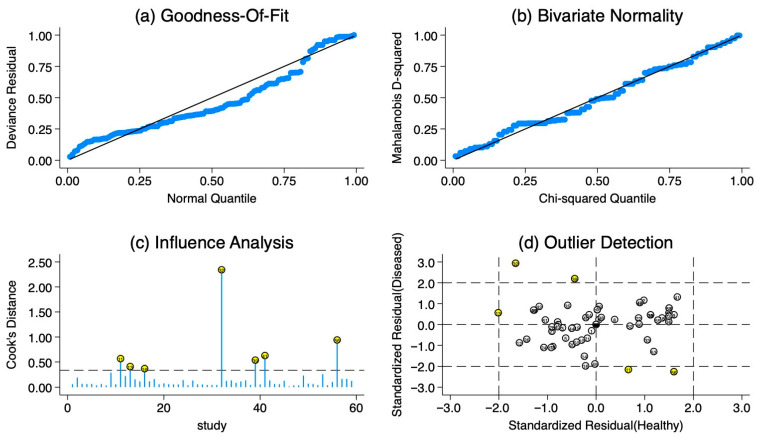
Sensitivity analysis. (**a**) Goodness-of-fit; (**b**) bivariate normality; (**c**) influence analysis; (**d**) outlier detection.

The sensitivity analysis is shown in Figure 5. The goodness-of-fit and bivariate normal distribution analyses indicated that the bivariate mixed-effects model was appropriate for this meta-analysis (Figure 5a,b). Outlier detection suggested that observations 11, 13, 32, 39, and 56 might contribute to the observed heterogeneity (Figure 5c,d).

#### 3.2.2. Subgroup Analysis and Meta-Regression

**Figure 6:**
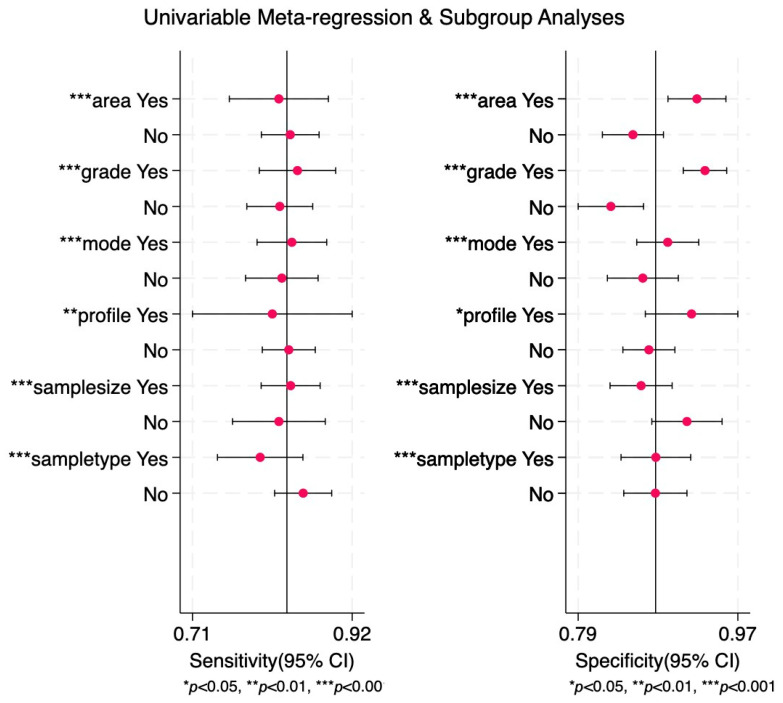
Univariable meta-regression and subgroup analyses for diagnosis of miRNAs in glioma. Variables were coded as follows: area (yes: other, no: Asia); grade (yes: high-grade glioma, no: low-grade glioma); mode (yes: upregulation, no: downregulation); profile (yes: cluster, no: single miRNA); sample size (yes: ≥100 patients, no: <100 patients); sample type (yes: plasma, no: serum); *p* < 0.05: statistically significant (*); *p* < 0.01: moderately significant (**); *p* < 0.001: strong statistical significance (***).

To identify possible sources of heterogeneity, we conducted subgroup and meta-regression analyses based on study region, glioma grade, regulation mode, profile, sample size, and sample type (Table 3 and Figure 6).

In the subgroup analysis, studies carried out in Asian countries demonstrated slightly lower diagnostic accuracy compared with those performed in other regions (sensitivity [0.84] vs. [0.82], specificity [0.85] vs. [0.93], PLR [5.6] vs. [12.5], NLR [0.19] vs. [0.19], DOR [29] vs. [66], and AUC [0.91] vs. [0.94]).

High-grade glioma demonstrated higher diagnostic accuracy compared to low-grade glioma in all the parameters: sensitivity [0.84] vs. [0.82], specificity [0.94] vs. [0.82], PLR [14.4] vs. [4.7], NLR [0.17] vs. [0.21], DOR [85] vs. [22], and AUC [0.95] vs. [0.89]. The AUC value for the high-grade glioma category was the highest [0.95] among all the subgroups, and the difference in the AUC values for the type of glioma subgroup was by far the most striking, suggesting that miRNAs have very strong potential for the diagnosis of high-grade glioma, which is important due to the lack of non-invasive diagnostic biomarkers for the disease that is usually detected at the late stages.

Additionally, miRNA clusters achieved higher diagnostic accuracy than single miRNAs (sensitivity [0.82] vs. [0.83], specificity [0.92] vs. [0.87], PLR [9.9] vs. [6.3], NLR [0.20] vs. [0.19], DOR [50] vs. [32], and AUC [0.94] vs. [0.92]).

Studies reporting upregulation of miRNAs showed superior performance compared with those focused on downregulation (sensitivity [0.84] vs. [0.82], specificity [0.90] vs. [0.86], PLR [8.3] vs. [5.7], NLR [0.17] vs. [0.20], DOR [48] vs. [28], and AUC [0.94] vs. [0.91]).

The sample type analysis revealed that serum-based studies demonstrated higher diagnostic accuracy than plasma-based studies (sensitivity [0.85] vs. [0.80], specificity [0.88] vs. [0.87], PLR [7.1] vs. [6.3], NLR [0.17] vs. [0.23], DOR [43] vs. [27], and AUC [0.93] vs. [0.91])

Significant differences were also observed between studies with smaller sample sizes (<100) compared with larger cohorts (≥100). Smaller studies showed several higher pooled estimates (sensitivity [0.83] vs. [0.84], specificity [0.95] vs. [0.86], PLR [17.0] vs. [5.9], NLR [0.18] vs. [0.19], DOR [94] vs. [31], and AUC [0.94] vs. [0.91]).

**Figure 7:**
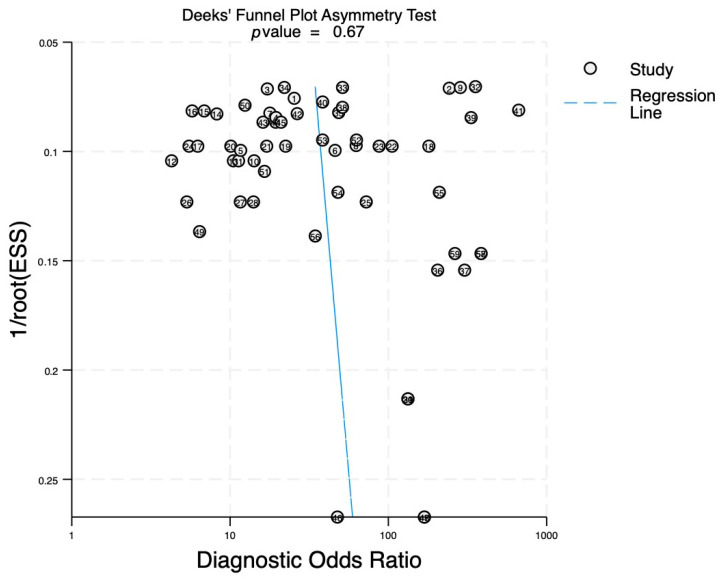
Deeks’ funnel plot asymmetry test for bias assessment.

A Deeks’ funnel plot was applied to assess publication bias (Figure 7), yielding a *p*-value of 0.67, showcasing that there is no evidence of statistically significant bias among the included studies. However, the Deeks’ funnel plot suggests that small studies might influence the results by overestimating the diagnostic performance. Thus, for future studies and clear conclusions regarding the diagnostic potential of miRNAs, studies with larger sample sizes are required to obtain a true estimation of the potential of miRNAs as a diagnostic tool for glioma.

**Figure 8:**
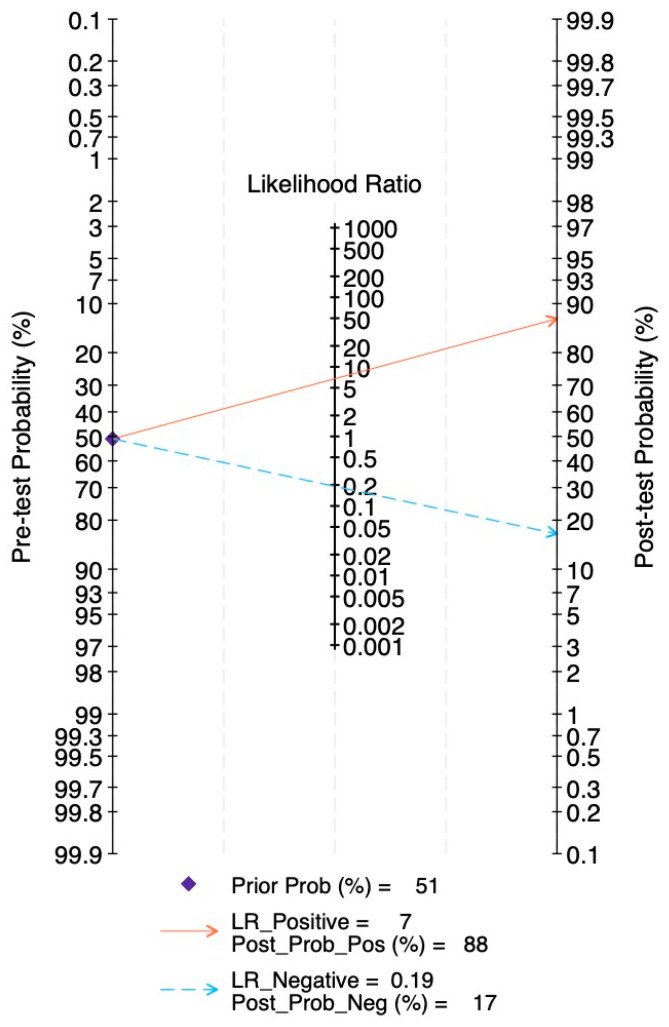
Fagan’s nomogram.

The Fagan’s nomogram (Figure 8) illustrated the clinical value of circulating miRNAs. With a pre-test probability equal to 51% and PLR equal to 6.7 (95%CI: 5.4–8.5), the post-test probability of a true positive increased to 88%, whereas the NLR of 0.19 (95%CI: 0.16–0.23) reduced the probability of disease in test-negative patients to 17%. These results support the clinical diagnostic potential of circulating miRNAs in glioma.

When the small Géczi D. et al. study [26] was excluded from the analysis, PLR became equal to 6.5 (95%CI: 5.2–8.2), NLR to 0.2 (95%CI: 0.16–0.24), and DOR to 33 (95%CI: 0.16–0.24). The observed decrease in DOR from 35 (95%CI: 25–50) with the study to 33 without the study suggests that small-number studies can have an effect on the diagnostic parameters, and caution should be taken while interpreting the results.

### 3.3. Discussion

Glioma is a tumor that originates in the brain, specifically in glial cells that surround and support neurons, astrocytes, oligodendrocytes and ependymal cells. The WHO 2021 classification distinguishes three main types of diffuse glioma in adults: astrocytoma, oligodendroglioma, and glioblastoma.

Glioblastoma (GBM) is the most aggressive and common primary malignant brain tumor in adults. It is classified as a WHO Grade IV glioma. GBM is characterized by high cellular heterogeneity, aggressive invasion into surrounding brain tissue, and resistance to therapy. The current treatments include maximal surgical resection, followed by radiotherapy and concurrent temozolomide (TMZ) chemotherapy [56]. However, due to the highly infiltrative nature of GBM, complete tumor resection is nearly impossible, which contributes to rapid tumor recurrence after surgery. The limited efficacy of the existing treatments highlights the urgent need for novel biomarkers for early tumor detection.

Glioma cell physiology depends on regulatory mechanisms, including the ones regulated by microRNAs. MicroRNAs are a class of non-coding RNAs that participate in regulations of gene expression at the posttranscriptional level. MicroRNAs are transcribed into pri-miRNAs by RNA polymerase II. Drosha, an RNase III enzyme, cleaves the pri-miRNAs to produce a pre-miRNA molecule approximately 85 nucleotides long [13]. After successful movement into the cell cytoplasm using the Exportin 5 molecule coupled with RanGTP, they are recognized by Dicer, which processes pre-miRNAs into mature miRNAs [57]. Mature miRNAs bind to argonaute proteins to form RNA-induced silencing complexes (RISCs), which bind to messenger RNAs and prevent translation by either destroying the mRNA molecule or temporarily silencing it. The target mRNA is recognized by complementarity regions. With an average length of mature miRNAs being ~22 nucleotides, a single miRNA can have multiple targets.

miRNAs play critical roles in tumor initiation, progression, and metastasis. Dysregulation of miRNAs is frequently observed in various cancers, including gliomas, where they can act as either oncogenic miRNAs (oncomiRs) or tumor suppressor miRNAs. OncomiRs work to promote tumor growth by suppressing tumor suppressor genes. Examples include miR-21, which is overexpressed in GBM, targeting tumor suppressors like Phospatase and Tensin Homolog (PTEN) and Programmed Cell Death Protein 4 (PDCD4) [58], leading to enhanced proliferation and invasion, while miR-155 promotes immune evasion by downregulating pro-inflammatory mediators [59]. On the other hand, tumor suppressor miRNAs are downregulated in GBM, leading to loss of growth inhibition. For instance, miR-34a targets oncogenes like Notch1 and Bcl-2, promoting apoptosis [60], while miR-124 suppresses glioma cell proliferation and invasion [61]. miRNA profiling studies have identified specific miRNA signatures associated with GBM development and progression.

**Figure 9:**
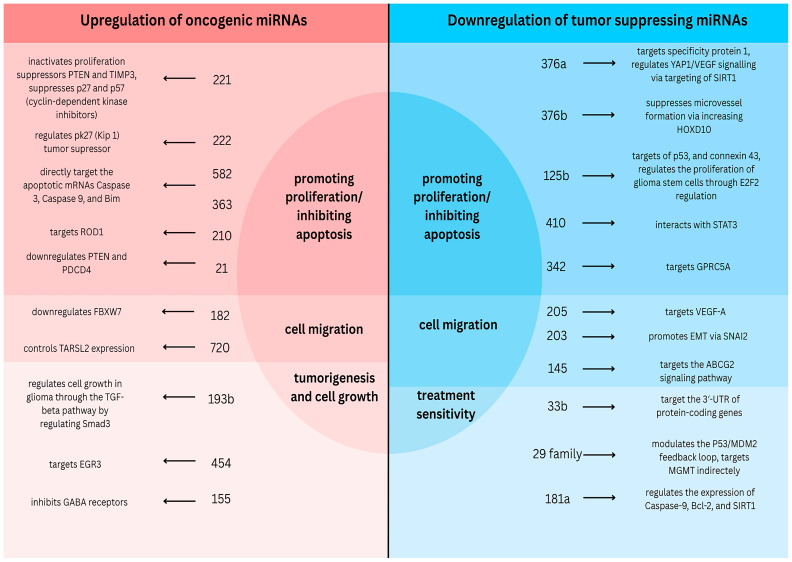
Modes of action of miRNAs analyzed in the paper. Glioma dysregulated in miRNAs could be classified into two classes—oncogenic and tumor-suppressing. Both act on similar pathways but in different directions. miRNAs analyzed in the current meta-analysis are summarized.

Figure 9 presents miRNAs attributed to the development of glioma and other cancers. It summarizes the mechanisms of oncogenic miRNAs that are upregulated and tumor suppressor miRNAs that are typically downregulated.

As miRNAs in gliomas can be classified as oncogenic and tumor suppressing, the mechanisms of their effect can be further classified into different categories. For example, such oncogenic miRNAs as miRNA-221, -222, -582, -363, -210, and -21 generally promote proliferation and/or inhibit apoptosis of tumor cells. They affect tumor suppressors like PTEN (miRNA-221 and miRNA-21) [58,62], deactivate the apoptotic mechanisms by targeting apoptotic mRNAs Caspase-3, Caspase-9, and Bim (miRNA-582 and miRNA-363) [63] or downregulating Programmed Cell Death Protein 4 (miRNA-21) [58], and regulate the proliferation of tumor cells and apoptosis by targeting ROD1 (miRNA-210) [64]. Similar effects can be achieved due to the downregulation of several tumor-suppressing miRNAs. Thus, miRNA-376a inhibits cell proliferation and invasion in GBM by directly targeting Specificity Protein 1 [65], while, in glioma, a similar effect together with inhibition of angiogenesis is achieved via the regulation of YAP1/VEGF signaling through targeting SIRT1 [66]. miRNA-376b also influences angiogenesis; however, its mechanism of operation is based on increasing HOXD10 content [67]. Thus, downregulation of this miRNA will lead to adverse effects and tumor-progressive growth. miRNA-125b similarly impacts glioma stem cell proliferation by targeting p53 [68], connexin43 [69], and regulating E2F2 [70]. miRNA-410 triggers apoptosis by its interaction with STAT3 mRNA [71]; thus, its lack leads to a lowered apoptosis rate and, consequently, tumor proliferation. Inhibition of glioma cell proliferation is also affected by miRNA-342, which targets GPRC5A [72].

Apart from the life cycle of cancer cells, miRNAs also affect cell migration, with oncogenic miRNAs typically promoting it, whereas tumor-suppressing miRNAs inhibit it. Thus, in glioma cells, expression of miRNA-182 leads to downregulation of FBXW7 protein, which results in promotion of cell proliferation and cell migration [73], while miRNA-720 controls TARSL2 (threonyl-tRNA synthetase like-2) expression, which leads to increased cell migration and invasion [74]. Among downregulated tumor-suppressing miRNAs, cell migration is influenced by miRNA-205, which affects VEGF-A [75], and miRNA- 203, whose downregulation promotes epithelial mesenchymal transition via regulating SNAI2 (also known as slug) [76]. Overexpression of miRNA-145 efficiently inhibits cell migration and invasion of glioma stem cells via its impact on the ABCG2 (ATP-binding cassette transporter protein) signaling pathway [77,78]; thus, downregulation of this miRNA leads to increased cell migration and invasion.

Tumorigenesis and cell growth are affected by various oncogenic miRNAs, including miRNAs-193b, -454, -222, and -155. The former, miRNA-193b, regulates cancer cell growth via the TGF-beta pathway by targeting Smad3 [79]. The target of miRNA-454 is EGR3 [80]. A similar effect is achieved by miRNA-155, which inhibits γ-aminobutyric acid (GABA) receptors [81]. miRNA-155 also induces proliferation of glioma cells through the PI3K/Akt pathway [82].

Among tumor-suppressing miRNAs, some affect treatment sensitivity, and their downregulation has a negative impact on treatment efficiency. Thus, miRNA-33b plays a significant role in inducing apoptosis of cancer cells via targeting the three-prime untranslated region (3′-UTR) of protein-coding genes that are connected with the resistance of cancer cells to such anticancer drugs as cisplatin, docetaxel, bortezomib, paclitaxel, and daunorubicin [83]. The family of miRNAs-29 sensitizes glioma cells towards temozolomide through modulation of the p53/MDM2 feedback loop, where p53 activates cell death and MDM2 (mouse double minute 2) acts as its inhibitor [84,85]. Finally, miRNA-181a sensitizes cancer cells to radiation treatment through its impact on apoptosis regulator Bcl2; it increases sub-G1 cell cycle arrest and induction of apoptosis by carmustine through regulation of the expression genes, including Caspase-9, Bcl-2, and SIRT1 [86].

Considering the involvement of miRNAs in tumor pathogenesis, it does not come as a surprise that they were considered as biomarkers for different cancers [87,88,89]. miRNAs were investigated as potential biomarkers for melanoma [90], Alzheimer’s disease and frontotemporal dementia [91], as well as cardiovascular diseases [92]. The benefits of miRNAs as potential biomarkers for early diagnostics and treatment strategy selection include non-invasive detection of the disease even before symptoms appear. miRNAs are differentially expressed in different grades of glioma and can be associated with patient survival. However, drawbacks need to be considered when implementing miRNA-based diagnostics of glioma. They include difficulty in reproducing the results, inconsistent sample collection and miRNA’s susceptibility to degradation.

In the current meta-analysis, we explored the diagnostic potential of freely circulating miRNAs detected in the blood of patients diagnosed with glioma. Overall, 31 original papers were analyzed, with 59 datapoints representing 2299 glioma patients and 1719 healthy controls. We conducted a meta-analysis, and we report here high values for pooled sensitivity equal to 0.83, pooled specificity equal to 0.88, as well as AUC equal to 0.92. The reported values suggest that miRNAs can be used as diagnostic biomarkers for glioma detection. A future perspective could include the identification of the most upregulated/downregulated miRNAs, with a subsequent evaluation of their mechanisms of action.

The question of miRNAs’ role in glioma diagnostics was addressed in several published papers [52,53,54]. The most recent one is by Hasani et al. (2024) [55]. Comparing the key parameters, it is worth noting that our reported values for sensitivity, specificity, as well as AUC are higher than the values reported by Hasani et al. [55]: 0.83 vs. 0.821 for sensitivity, 0.88 vs. 0.831 for specificity, and 0.92 vs. 0.893 for AUC. There is a difference, however, in the analyzed samples between these two studies. Within our study, we focused on the reports on miRNAs freely circulating in blood, whereas Hasani et al., evaluated miRNAs in blood as well as in brain tissue and cerebrospinal fluid (CSF). Considering that, in the Hasani publication, the overall number of glioma patients (3111) and controls (2045) is higher compared to our pool, it is possible that, with an increase in the sample size, the values for sensitivity, specificity, and AUC will decrease.

Within our study, most of the reports included in the meta-analysis were of Asian origin, which represents one of the possible limitations as the key parameters might not represent the global tendency. Another possible limitation of the present study is the high degree of heterogeneity within the included studies. The potential sources of heterogeneity could come from differences in sample size, methodological differences in microRNA isolation, cDNA synthesis, and the detection approach, where different normalization controls are used. Age differences and sex differences in the patients and control groups within the analyzed papers could also potentially be a source of heterogeneity. We also included studies addressing freely circulating miRNAs in the blood of patients of all glioma grades (from I to IV). This could potentially contribute to heterogeneity as our subgroup analysis indicates that diagnostic parameters for HGG are higher compared to LGG, suggesting that miRNAs might be better diagnostic markers for glioma of higher grades. In the future, with a collection of more experimental data, it might be possible to look at the diagnostic potential of miRNAs for a particular glioma type.

In conclusion, the conducted study suggests that miRNAs freely circulating in blood hold the potential for glioma diagnostics. However, the high level of heterogeneity implies that the present results should be considered as exploratory.

## 4. Conclusions

Glioma is a devastating rapidly progressing disease with a high toll on the life of individuals with the disease and their caregivers. It is characterized by limited diagnostic and treatment options. The currently available diagnostic tools allow the detection of the disease only at late stages when treatment options are limited. Thus, high demand exists for reliable early diagnostics of glioma. The biomarkers present in the blood of patients should be considered to make glioma diagnostics robust and enable them to be conducted at earlier stages. The present study evaluated miRNAs present in the blood as diagnostic biomarkers of glioma. Our results suggest that

- The statistical analysis yielded high values for sensitivity at 0.83, selectivity at 0.88, and AUC at 0.92, suggesting good diagnostic potential of miRNAs in glioma;
- High heterogeneity was observed between the pooled articles, which limits the conclusions of the study and necessitates standardization of miRNA extraction and detection, as well as of normalization control. Further validation of miRNAs is needed before clinical application;
- Studies exploring the diagnostic potential of glioma-associated miRNAs are biased by insufficient disclosure of patient selection criteria and blinding methods in conducting the index test and reference standard for glioma diagnostics;
- Oncogenic microRNAs with the suggested mechanisms, such as miR-221, miR-222, miR-582-5p, miR-363, miR-210, miR-21, miR-182, miR-720, miR-193b, miR-454, and miR-155, are upregulated in the blood of glioma patients;
- Tumor suppressor microRNAs with the suggested mechanisms, such as miR-376a, miR-376b, miR-125b, miR-410, miR-181b, miR-342, miR-205, miR-203, miR-145, miR-33b, miR-100, miR-29, and miR-181a, are downregulated in the blood of glioma patients.

Overall, despite finding high diagnostic values for miRNAs in glioma, the high heterogeneity in the assessed studies suggests that the conclusions should be viewed as exploratory at present.

## Figures and Tables

**Table 1:** Characteristics of the included studies.

Study	Year	Country	Sample Source	Patient Sample Size	Control Sample Size
Ali E. [19]	2025	Egypt	Serum	25	20
Barut Z. [20]	2023	Turkey	Serum	25	25
Billur D. [21]	2022	Turkey	Serum	35	36
Bustos M. [22]	2022	USA	Plasma	45	73
Chen J. [23]	2017	China	Serum	70	30
Chen P. [24]	2020	China	Plasma	122	60
Donofrio C. [25]	2025	Italy	Plasma	23	32
Géczi D. [26]	2021	Hungary	Plasma	6	6
Huang Q. [27]	2017	China	Serum	100	50
Lai N.-S. [28]	2015	China	Serum	136	50
Ohno M. [29]	2019	Japan	Serum	57	114
Qi Y. [30]	2020	China	Serum	128	62
Shao N. [31]	2015	China	Plasma	70	70
Sun J. [32]	2015	China	Serum	151	53
Swellam M. [33]	2019	Egypt	Serum	20	20
Tang Y. [34]	2017	China	Plasma	74	74
Wang J. [35]	2019	China	Serum	100	100
Wang Q. [36]	2012	China	Plasma	10	10
Wei X. [37]	2016	China	Serum	33	33
Wu J. [38]	2022	China	Plasma	38	85
Wu J.H. [39]	2014	China	Serum	83	69
Xiao Y. [40]	2016	China	Plasma	112	54
Xu W. [41]	2017	Russia	Plasma	47	45
Yang C. [42]	2013	China	Serum	133	80
Yue X. [43]	2016	China	Serum	64	45
Zhang H. [44]	2019	China	Serum	95	60
Zhang R. [45]	2016	China	Plasma	50	51
Zhang Y. [46]	2019	China	Serum	117	50
Zhao S. [47]	2016	China	Serum	118	84
Zhi F. [48]	2014	China	Serum	90	110
Zhu M. [49]	2019	China	Serum	122	68

**Table 2:** The miRNA characteristics in the included studies.

Entry	Type of miRNA	Mode of Expression	Grade or Type	Sensitivity	Specificity	AUC	Study
1	miR-29a	up	GBM	0.88	1.00	0.978	Ali E. [19]
2	miR-106a	up	GBM	0.92	1.00	0.956	
3	miR-200a	up	GBM	0.92	1.00	0.98	
4	miR-22-3p	down	GBM	0.417	1.00	0.674	Barut Z. [20]
5	miRNA-582-5p’	up	GBM	0.844	0.97	0.938	Billur D. [21]
6	miRNA-363	up	GBM	0.893	0.861	0.951	
7	miR-5739	up	GBM	0.622	0.959	0.848	Bustos M. [22]
8	miR-3180-3p	up	GBM	0.832	0.932	0.881	
9	miR-203	down	GBM	0.8586	0.7336	0.862	Chen J. [23]
10	miR-720	up	Grades I–IV	0.713	0.833	0.773	Chen P. [24]
11	miR-34a-5p	up	GBM	0.478	0.875	0.664	Donofrio C. [25]
12	miR-433-3p	up	GBM	0.92	0.96	0.98214	Géczi D. [26]
13	miR-29a-3p	up	GBM	0.92	0.96	0.98214	
14	miR-195-5p	up	GBM	0.88	0.96	0.9704	
15	miR-376c	down	Grades I–IV	0.90	0.70	0.837	Huang Q. [27]
16	miR-376a	down	Grades I–IV	0.81	0.82	0.872	
17	miR-376b	down	Grades I–IV	0.82	0.78	0.890	
18	miR-210	up	Grades I–IV	0.9127	0.725	0.927	Lai N.-S. [28]
19	combination of miR-4763-3p,	up	diffuse glioma	0.95	0.97	0.99	Ohno M. [29]
	miR-1915-3p,						
	miR-3679-5p						
20	miR-33b	down	Grades I–IV	0.867	0.855	0.883	Qi Y. [30]
21	miR-454-3p	up	Grades I–IV	0.9905	0.8286	0.9063	Shao N. [31]
22	miR-128	down	Grades I–IV	0.8675	0.8868	0.9095	Sun J. [32]
23	miR-221	up	GBM	0.90	1.00	0.925	Swellam M. [33]
24	miR-222	up	GBM	0.85	1.00	0.957	
25	miR-122	down	Grades I–IV	0.919	0.811	0.939	Tang Y. [34]
26	miR-214	up	overall glioma	0.90	0.71	0.885	Wang J. [35]
27	miR-214	up	HGG	0.7258	0.95	0.909	
28	miR-214	up	LGG	1.00	0.64	0.847	
29	miR-21	up	GBM	0.90	1.00	0.93	Wang Q. [36]
30	miR-128	down	GBM	0.90	1.00	1.0	
31	miR-342-3p	down	GBM	0.90	1.00	1.0	
32	miR-125b	down	Grade II	0.8182	0.7576	0.868	Wei X. [37]
33	miR-125b	down	Grades I–IV	0.7879	0.7576	0.839	
34	miR-125b	down	Grade I	0.7273	0.6667	0.691	
35	miR-125b	down	Grades III–IV	0.9091	0.8788	0.959	
36	miR-410	down	LGG	0.657	0.741	0.67	Wu J. [38]
37	miR-181b	down	HGG	0.931	0.887	0.94	
38	miR-410	down	HGG	0.868	0.9421	0.97	
39	miR-181a	down	LGG	0.7334	0.860	0.83	
40	miR-181b	down	LGG	0.6821	0.8275	0.78	
41	miR-155	up	HGG	0.823	0.841	0.92	
42	miR-181a	down	HGG	0.875	0.967	0.97	
43	miR-155	up	LGG	0.667	0.769	0.68	
44	miR-29 family	down	LGG	0.49	0.85	0.66	Wu J.H. [39]
45	miR-29 family	down	Grades I–IV	0.685	0.773	0.74	
46	miR-182	up	Grades I–IV	0.585	0.852	0.778	Xiao Y. [40]
47	miR-17	up	Grades I–IV	0.893	0.553	0.787	Xu W. [41]
48	miR-130a	up	Grades I–IV	0.702	0.652	0.720	
49	miR-10b	up	Grades I–IV	0.446	0.936	0.721	
50	combination of 3 miRNAs (miR-17, miR-130a, and miR-10b)	up	Grades I–IV	0.723	0.851	0.872	
51	combination of 7 miRNAs (miR-15b *, miR-23a, miR-133a, miR-150 *, miR-197, miR-497, miR-548b-5p)	down	Grades II–IV	0.88	0.9787	0.972	Yang C. [42]
52	miR-205	down	Grades I–IV	0.863	0.922	0.935	Yue X. [43]
53	miR-100	down	GBM	0.8333	0.7789	0.839	Zhang H. [44]
54	miR-222	up	glioma	0.857	0.875	0.92	Zhang R. [45]
55	miR-221	up		0.735	0.80	0.84	
56	miR-145-5p	down	GBM	0.846	0.78	0.895	Zhang Y. [46]
57	miR-451a	down	glioma	0.814	0.797	0.816	Zhao S. [47]
58	combination of 9 miRNAs (miR-15b-5p, miR-16-5p, miR-19a-3p, miR-19b-3p, miR-20a-5p, miR-106a-5p, miR-130a-3p, miR-181b-5p, miR-208a-3p)	down	Grades II–IV	0.933	0.945	0.9722	Zhi F. [48]
59	miR-193b	up	Grades I–IV	0.795	0.868	0.903	Zhu M. [49]

’ Red and blue fonts highlight upregulated and downregulated miRNAs, respectively, mechanisms of which are discussed in the paper.

**Table 3:** Subgroup analysis. (Sen: sensitivity; Spe: specificity; PLR: positive likelihood ratio; NLR: negative likelihood ratio; DOR: diagnostic odds ratio; AUC: area under the curve.)

Subgroups	No. Observations	Sen [95%CI]	Spe [95%CI]	PLR [95%CI]	NLR [95%CI]	DOR [95%CI]	AUC [95%CI]
Area							
Asia	40	0.84 [0.79–0.87]	0.85 [0.81–0.88]	5.6 [4.4–7.0]	0.19 [0.15–0.24]	29 [20–42]	0.91 [0.88–0.93]
Other	19	0.82 [0.75–0.87]	0.93 [0.88–0.976]	12.5 [6.6–23.9]	0.19 [0.13–0.27]	66 [26–166]	0.94 [0.92–0.96]
Grade							
High-grade	26	0.84 [0.79–0.88]	0.94 [0.90–0.97]	14.4 [8.4–24.8]	0.17 [0.13–0.23]	85 [44–167]	0.95 [0.93–0.97]
Low-grade	33	0.82 [0.77–0.87]	0.82 [0.79–0.86]	4.7 [3.8–5.8]	0.21 [0.16–0.28]	22 [15–33]	0.89 [0.86–0.92]
Mode							
Down	28	0.82 [0.78–0.86]	0.86 [0.82–0.89]	5.7 [4.5–7.4]	0.20 [0.16–0.26]	28 [18–43]	0.91 [0.88–0.93]
Up	31	0.84 [0.78–0.89]	0.90 [0.85–0.93]	8.3 [5.5–12.6]	0.17 [0.12–0.24]	48 [26–88]	0.94 [0.91–0.96]
Profile							
Single	53	0.83 [0.80–0.86]	0.87 [0.83–0.89]	6.3 [5.0–7.8]	0.19 [0.16–0.24]	32 [23–45]	0.92 [0.89–0.94]
Cluster	6	0.82 [0.65–0.91]	0.92 [0.84–0.96]	9.9 [4.3–22.7]	0.20 [0.09–0.42]	50 [11–230]	0.94 [0.92–0.96]
Sample Size							
<100	23	0.83 [0.75–0.88]	0.95 [0.87–0.98]	17.0 [6.1–47.5]	0.18 [0.12–0.27]	94 [28–322]	0.94 [0.91–0.96]
≥100	36	0.84 [0.80–0.87]	0.86 [0.82–0.88]	5.9 [4.7–7.2]	0.19 [0.15–0.24]	31 [21–44]	0.91 [0.89–0.94]
Sample Type							
Serum	32	0.85 [0.81–0.89]	0.88 [0.83–0.92]	7.1 [5.1–10.0]	0.17 [0.13–0.21]	43 [27–69]	0.93 [0.90–0.95]
Plasma	27	0.81 [0.74–0.85]	0.87 [0.83–0.91]	6.3 [4.6–8.5]	0.23 [0.17–0.31]	27 [16–46]	0.91 [0.88–0.93]

## Data Availability

No new data were created or analyzed in this study.

## Supplementary Materials

The following supporting information can be downloaded at https://www.mdpi.com/article/10.3390/ijms27041680/s1.

## Abbreviations

The following abbreviations are used in this manuscript:

GBM	Glioblastoma
HGG	High-grade glioma
LGG	Low-grade glioma
PLR	Positive likelihood ratio
NLR	Negative likelihood ratio
DOR	Diagnostic odds ratio
AUC	Area under the curve
