# Supplementary material for: Diagnostic Potential of Circulating miRNAs in Glioma: A Systematic Review and Meta-Analysis
Source: Int J Mol Sci. 2026 Feb 9;27(4):1680. doi: 10.3390/ijms27041680 (PMC12941128; doi:10.3390/ijms27041680)
Supplement: Supplementary file 1 [file ijms-27-01680-s001.zip › ijms-4038208-supplementary.pdf]

A

Study Name	Risk of Bias: Patient Selection	Risk of Bias: Index Test	Risk of Bias: Reference Standard	Risk of Bias: Flow and Timing	Applicability Concerns: Patient Selection	Applicability Concerns: Index Test	Applicability Concerns: Reference Standard
Ali E., 2025. [19]	Unclear	Unclear	Unclear	Unclear	Low	Low	Low
Barut Z., 2023 [20]	Unclear	Unclear	Unclear	Unclear	Low	Low	Low
Billur D., 2022 [21]	Unclear	Unclear	Unclear	Unclear	Low	Low	Low
Bustos M., 2022 [22]	Unclear	Unclear	Low	Low	Low	Low	Low
Chen J., 2017 [23]	Unclear	Unclear	High	Low	Low	Low	Low
Chen P., 2020 [24]	Low	Unclear	Low	Low	Low	Low	Low
Donofrio C., 2025 [25]	Unclear	Unclear	Low	Unclear	High	Low	Low
Géczi D., 2021 [26]	Unclear	High	Low	High	High	Low	Low
Huang Q., 2017 [27]	Unclear	Unclear	Unclear	Unclear	Low	Low	Low
Lai N.S., 2015 [28]	Unclear	Unclear	Unclear	Unclear	Low	Low	Low
Ohno M., 2019 [29]	Unclear	Unclear	Unclear	Low	Low	Low	Low
Qi Y., 2020 [30]	Unclear	High	Low	Low	Low	Low	Low
Shao N., 2015 [31]	Unclear	High	Low	Low	Low	Low	Low
Sun J., 2015 [32]	Unclear	Unclear	Low	High	Low	Low	Low
Swellam M., 2019 [33]	Unclear	Unclear	Unclear	Low	Low	Low	Low
Tang Y., 2017 [34]	Unclear	Unclear	Low	Low	Low	Low	Low
Wang J., 2019 [35]	Unclear	High	Unclear	Low	Low	Low	Low
Wang Q., 2012 [36]	Unclear	High	Low	Low	Low	Low	Low
Wei X., 2016 [37]	Unclear	Unclear	Low	Low	Low	Low	Low
Wu J., 2022 [38]	Unclear	Unclear	Low	Low	Low	Low	Low
Wu J.H., 2014 [39]	Unclear	Unclear	Unclear	Low	Low	Low	Low
Xiao Y., 2016 [40]	Unclear	Unclear	Unclear	Low	Low	Low	Low
Xu W., 2017 [41]	Unclear	Unclear	Low	Low	Low	Low	Low
Yang C., 2013 [42]	Unclear	High	Unclear	Unclear	Low	Low	Low
Yue X., 2016 [43]	Unclear	High	Low	Low	Low	Low	Low
Zhang H., 2019 [44]	Unclear	Unclear	Unclear	Unclear	Low	Low	Low
Zhang R., 2016 [45]	Unclear	High	Unclear	Unclear	Low	Low	Low
Zhang Y., 2019 [46]	Unclear	Unclear	Unclear	Low	Low	Low	Low
Zhao S., 2016 [47]	Unclear	High	Unclear	Low	Low	Low	Low
Zhi F., 2014 [48]	Unclear	Low	Unclear	High	Low	High	Low
Zhu M., 2019 [49]	Low	Unclear	Unclear	Low	Low	Low	Low

B

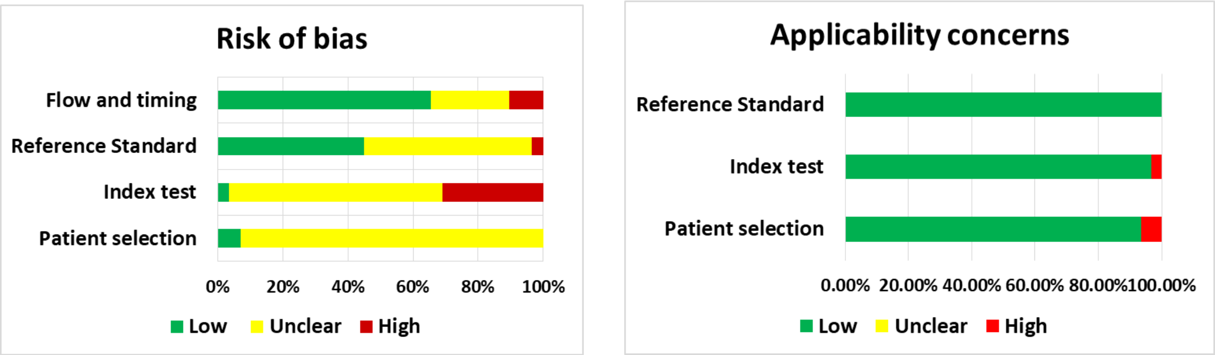

Supplementary Figure S1. QUADAS-2 evaluations included in meta-analysis papers: A—assessment of individual papers; B—summary of the assessment.

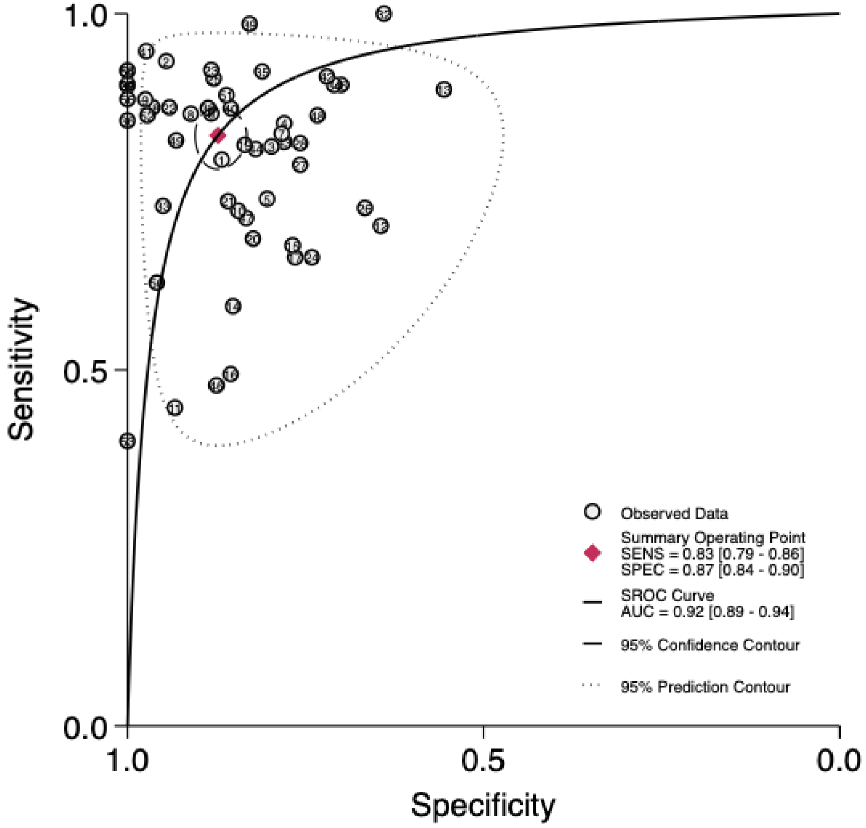

Supplementary Figure S2. SROC curve with excluded Géczi D. et al. [26] study.

Supplementary Table S1. Derived values of TP, TN, FP, and FN used for analysis.

Observation #	N Patients	N Controls	TP	FP	FN	TN	Sensitivity Calculated	Sensitivity Reported	Specificity Calculated	Specificity Reported
1	25	20	22	0	3	20	88.00%	88.00%	100.00%	100.0%
2	25	20	23	0	2	20	92.00%	92.00%	100.00%	100.0%
3	25	20	23	0	2	20	92.00%	92.00%	100.00%	100.0%
4	25	25	10	0	15	25	40.00%	41.70%	100.00%	100.0%
5	35	36	30	1	5	35	85.71%	84.40%	97.22%	97.0%
6	35	36	31	5	4	31	88.57%	89.30%	86.11%	86.1%
7	45	73	28	3	17	70	62.22%	62.20%	95.89%	95.9%
8	45	73	37	5	8	68	82.22%	83.20%	93.15%	93.2%
9	70	30	60	8	10	22	85.71%	85.86%	73.33%	73.4%
10	122	60	87	10	35	50	71.31%	71.30%	83.33%	83.3%
11	23	32	11	4	12	28	47.83%	47.80%	87.50%	87.5%
12	6	6	6	0	0	6	100.00%	92.00%	100.00%	96.0%
13	6	6	6	0	0	6	100.00%	92.00%	100.00%	96.0%
14	6	6	5	0	1	6	83.33%	88.00%	100.00%	96.0%
15	100	50	90	15	10	35	90.00%	90%	70.00%	70.0%
16	100	50	81	9	19	41	81.00%	81%	82.00%	82.0%
17	100	50	82	11	18	39	82.00%	82%	78.00%	78.0%
18	136	50	124	14	12	36	91.18%	91.27%	72.00%	72.5%
19	57	114	54	3	3	111	94.74%	95%	97.37%	97.0%
20	128	62	111	9	17	53	86.72%	86.70%	85.48%	85.5%
21	70	70	69	12	1	58	98.57%	99.05%	82.86%	82.9%
22	151	53	131	6	20	47	86.75%	86.75%	88.68%	88.7%
23	20	20	18	0	2	20	90.00%	90%	100.00%	100.0%
24	20	20	17	0	3	20	85.00%	85%	100.00%	100.0%
25	74	74	68	14	6	60	91.89%	91.90%	81.08%	81.1%
26	100	100	90	29	10	71	90.00%	90.00%	71.00%	71.0%
27	100	100	73	5	27	95	73.00%	72.58%	95.00%	95.0%
28	100	100	100	36	0	64	100.00%	100.00%	64.00%	64.0%
29	10	10	9	0	1	10	90.00%	90%	100.00%	100.0%
30	10	10	9	0	1	10	90.00%	90%	100.00%	100.0%
31	10	10	9	0	1	10	90.00%	90%	100.00%	100.0%
32	33	33	27	8	6	25	81.82%	81.82%	75.76%	75.8%
33	33	33	26	8	7	25	78.79%	78.79%	75.76%	75.8%
34	33	33	24	11	9	22	72.73%	72.73%	66.67%	66.7%

35	33	33	30	4	3	29	90.91%	90.91%	87.88%	87.9%
36	38	85	25	22	13	63	65.79%	65.70%	74.12%	74.1%
37	38	85	35	10	3	75	92.11%	93.10%	88.24%	88.7%
38	38	85	33	5	5	80	86.84%	86.80%	94.12%	94.2%
39	38	85	28	12	10	73	73.68%	73.34%	85.88%	86.0%
40	38	85	26	15	12	70	68.42%	68.21%	82.35%	82.8%
41	38	85	31	14	7	71	81.58%	82.30%	83.53%	84.1%
42	38	85	33	3	5	82	86.84%	87.50%	96.47%	96.7%
43	38	85	25	20	13	65	65.79%	66.70%	76.47%	76.9%
44	83	69	41	10	42	59	49.40%	49%	85.51%	85.0%
45	83	69	56	16	27	53	67.47%	68%	76.81%	77.3%
46	112	54	66	8	46	46	58.93%	58.50%	85.19%	85.2%
47	47	45	42	20	5	25	89.36%	89.30%	55.56%	55.3%
48	47	45	33	16	14	29	70.21%	70.20%	64.44%	65.2%
49	47	45	21	3	26	42	44.68%	44.60%	93.33%	93.6%
50	47	45	34	7	13	38	72.34%	72.30%	84.44%	85.1%
51	133	80	117	2	16	78	87.97%	88.00%	97.50%	97.9%
52	64	45	55	4	9	41	85.94%	86.30%	91.11%	92.2%
53	95	60	79	13	16	47	83.16%	83.33%	78.33%	77.9%
54	50	51	43	6	7	45	86.00%	85.70%	88.24%	87.5%
55	50	51	37	10	13	41	74.00%	73.50%	80.39%	80.0%
56	117	50	99	11	18	39	84.62%	84.60%	78.00%	78.0%
57	118	84	96	17	22	67	81.36%	81.40%	79.76%	797%
58	90	110	84	6	6	104	93.33%	93.30%	94.55%	94.5%
59	122	68	97	9	25	59	79.51%	79.50%	86.76%	86.8%
